# Fluctuation of Acquired Resistance Mutations and Re-Challenge with EGFR TKI in Metastatic NSCLC: A Case Report

**DOI:** 10.3390/curroncol30100640

**Published:** 2023-09-28

**Authors:** Markus Falk, Stefanie Schatz, Fabian P. M. Reich, Stefanie Schmidt, Marco Galster, Markus Tiemann, Joachim H. Ficker, Wolfgang M. Brueckl

**Affiliations:** 1 Institute for Hematopathology Hamburg, Fangdieckstraße 75A, 22547 Hamburg, Germany; falk@hp-hamburg.de (M.F.); schatz@hp-hamburg.de (S.S.); schmidt@hp-hamburg.de (S.S.); mtiemann@hp-hamburg.de (M.T.); 2 Department of Respiratory Medicine, Allergology and Sleep Medicine, Paracelsus Medical University, General Hospital Nuremberg, Ernst-Nathan-Str. 1, 90419 Nuremberg, Germany; fabian.reich@klinikum-nuernberg.de (F.P.M.R.); joachim.ficker@klinikum-nuernberg.de (J.H.F.); 3 Department of Radiology, Paracelsus Medical University, General Hospital Nuremberg, Ernst-Nathan-Str. 1, 90419 Nuremberg, Germany; marco.galster@klinikum-nuernberg.de

**Keywords:** disease monitoring, *EGFR*, hybrid capture NGS, osimertinib, liquid biopsy

## Abstract

Osimertinib has become the preferred first-line therapy for epidermal growth factor receptor (*EGFR)* mutation-positive metastatic non-small cell lung cancer (NSCLC) in recent years. Originally, it was approved for second-line treatment after epidermal growth factor receptor *EGFR* tyrosine kinase inhibitors (TKIs) of the first and second generations had failed and *EGFR* T790M had emerged as a mode of resistance. Osimertinib itself provokes a wide array of on- and off-target molecular alterations that can limit therapeutic success. Liquid biopsy ctDNA (circulating tumor DNA) analysis by hybrid capture (HC) next-generation sequencing (NGS) can help to identify alterations in a minimally invasive way and allows for the detection of common as well as rare resistance alterations. We describe a young female patient who was initially diagnosed with metastatic *EGFR* L858R-positive NSCLC. She received *EGFR* TKI therapy at different timepoints during the course of the disease and developed sequential *EGFR* resistance alterations (*EGFR* T790M and C797S). In the course of her disease, resistance alteration became undetectable, and the tumor was successfully rechallenged with the original first-generation *EGFR* TKI as well as osimertinib and altogether showed prolonged response despite a prognostically negative *TP53* alteration. To date, the patient has been alive for more than seven years, though initially diagnosed with a heavy metastatic burden.

## 1. Introduction

The incidence and mortality of lung cancer rank among the top three cancers worldwide. Accounting for 85% of the total number of lung cancer, non-small cell lung cancer (NSCLC) is a significant factor in human health [1]. Among different lung cancer histologies, adenocarcinomas, in contrast to squamous cell carcinomas, are often driven by a driver alteration [2]. Targeted therapy has led to a profound extension of overall survival (OS) in patients with stage IV NSCLC [3,4]. The most prevalent targetable mutation in NSCLC besides KRAS are epidermal growth factor receptor (*EGFR)* mutations, with a frequency of about 10–15% in the western world [1,5]. Since 2009, several *EGFR* TKIs have been approved for first-line treatment. TKIs of the first-generation (i.e., erlotinib) are reversible inhibitors that compete with ATP binding to the active site of *EGFR*. Second-generation TKIs (i.e., dacomitinib and afatinib) are irreversible inhibitors and target not only *HER1* (*EGFR*) but also other *HER* (human epidermal receptor) family members. T790M is the most common resistance mutation in these generations of TKIs. T790M leads to both steric hindrance and increased ATP affinity to mutant *EGFR* receptors, resulting in decreased efficacy of first- and second-generation *EGFR* TKI [6]. Based on the AURA trial, osimertinib, a third-generation *EGFR* TKI, was initially approved for second-line treatment of NSCLC that had progressed under TKI and developed a T790M mutation [7]. Later, with the success of the FLAURA trial, osimertinib complemented the portfolio of first-line TKIs, and at the same time, diminished the relevance of sequential TKI therapy due to superior survival data compared to other TKIs [8]. Resistance to osimertinib limits therapeutic success and can be heterogeneous in nature, including on- and off-target molecular alterations like *MET* and *EGFR* amplification or the *EGFR* C797X point mutation [9,10]. The C797S mutation in exon 20 prevents the third-generation *EGFR* TKI from forming covalent bonds in the tyrosine kinase region of *EGFR* [11]. Since some of the resistance mechanisms open up new therapeutic targets, genetic re-assessment of the tumor DNA should be performed at relapse under TKI therapy. Liquid biopsy has become an important alternative in this situation when additional tissue biopsies cannot be obtained or are refused by the patient. However, ctDNA is not reliably shed into the peripheral blood, which corresponds to tumor mass. In about 20% of cases, no corresponding tumor DNA is found in the blood, despite NSCLC stage IV disease [12,13]. It has been further observed that ctDNA analysis can predict resistance prior to imaging techniques and is more sensitive [14,15].

## 2. Case Report

We report the therapeutic course of a 45 year old female former smoker (12 pack years) who initially presented with oppressive headache and dizziness, leading to the diagnosis of NSCLC at the left hilus, including cerebral and osseous metastases. Clinical staging in June 2016 revealed cT2a Nx M1c (pul/cer/oss) UICC IVB disease, and subsequent pathological tissue evaluation indicated an *EGFR* L858R mutated adenocarcinoma. In July 2016, the patient received one cycle of palliative chemotherapy cisplatin/pemetrexed plus whole brain radiation (39.6 Gy) and radiation of sacral vertebra 9–11 (40 Gy) and 1–3 (38 Gy). Starting with chemotherapy was necessary because there was a clinical need for treatment and the mutational status was delayed. In August 2016, palliative TKI therapy with erlotinib was initiated because osimertinib had not been approved for first-line therapy in Europe by this time, resulting in partial remission through February until May 2017. In November 2017, moderate progress was documented, and a liquid biopsy was taken. CtDNA was extracted from plasma and analyzed for point mutations, gene amplifications, and translocations by HC NGS (sequencing platform Illumina NextSeq 500), revealing the original activating point mutation L858R (allele frequency (AF) 0.62%) combined with a T790M (AF 0.32%) resistance mutation and another *EGFR* point mutation, L838V (AF 0.57). The latter is regarded as a primary activating mutation and was reported to co-occur with L858R in TKI-naïve tumors as a compound mutation [16].

**Figure 1:**
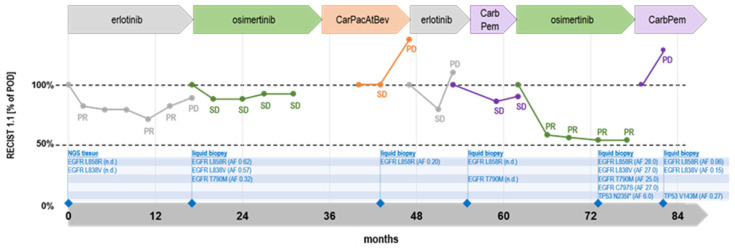
Chart of therapy sequence including type of therapy, RECIST data [%] and molecular result with allele frequency and type of analysis. n.d.: Not determined.

Therapy was subsequently switched to third-line osimertinib in February 2018 in line with clinical practice at the time. From June 2018 to May 2019, partial remission of the primary lung lesion was achieved with osimertinib. However, in parallel, newly arising osseous metastases required radiation of different anatomical sites, including the os ilium, femur, and shoulder. Starting in May 2019, tumor progress was observed in the left hilus and cerebrally. According to the results of the IMpower150 trial, atezolizumab/bevacizumab/paclitaxel/carboplatin were administered in July 2019 but resulted in stable disease for almost a year. In May 2020, a progressive disease was diagnosed regarding the primary and ulnar metastases. A liquid biopsy was taken and analyzed by NGS, and since only the L858R mutation was detectable but no resistance mutation (i.e., T790M), the patient was rechallenged with erlotinib, resulting in a moderate remission of the primary lesion and the pulmonary metastases until January 2021, when progressive disease at multiple sites was diagnosed and another liquid biopsy was taken. This time, due to poor ctDNA concentration, ctDNA analysis was conducted by cobas^®^ *EGFR* Mutation Test v2, and T790M was found in addition to the L858R. Due to the patient’s wishes she subsequently received carboplatin and pemetrexed as sixth-line treatment, followed by pemetrexed maintenance therapy leading to disease stabilization. However, kidney values worsened, leading to a rechallenge with osimertinib. In December 2021, significant partial remission was achieved that lasted about one year and was superseded by progressive disease. A liquid biopsy showed relatively high allele frequencies of the previously detected *EGFR* L858R (AF 27%), T790M (AF 25%), and L838V (AF 27%). Additionally, an *EGFR* C797S (AF 27%) was present and a pathogenic *TP53* point mutation N234I* (AF 6%). *EGFR* C797S is one of the most common modes of resistance to osimertinib treatment [9]. CtDNA was also mutated in *TP53,* showing a likely inactivating, pathogenic *TP53* mutation N235I* that leads to a premature translational stop. *TP53* mutations are not regarded as classical resistance mutations. The *TP53* mutation was not detected at the primary diagnosis or later on during the course of therapy. Since the allelic frequency of *TP53* N234I* (AF 6%) was low compared to the *EGFR* mutations found in the same liquid biopsy (AF 25–27%), it may indicate subclonal differentiation. Since both resistance mutations, *EGFR* T790M and C797S, occurred in cis (located on the same allele), TKI combination therapy was not regarded as expedient, and therefore carboplatin and pemetrexed were administered up to the time of the preparation of this manuscript. The latest liquid biopsy analysis (April 2023) revealed low allele frequencies of L858R (AF 0.06%), L838V (AF 0.15%), the absence of T790M and C797S, and again a new *TP53* variant V143M (AF 0.27), that was previously undetectable. At the same time, RECIST showed progressive disease under carboplatin/pemetrexed. Up to date (July 2023), the patient is treated with docetaxel and ramucirumab. Clinical and therapeutic follow-up as well as molecular diagnosis are summarized together with responses by RECIST in Figure 1.

## 3. Discussion

We describe a patient with stage IV NSCLC metastasized in many organs at diagnosis with a seven-year therapeutic course, including different generations of EGFR TKI. Despite an impressively supporting body of literature, molecular retesting at the time of progression under TKI treatment is still not a broadly applied clinical practice. Virtually all tumors that are treated with molecularly stratified therapies do relapse over time. Lung cancer has evolved as a model disease over the years, and detailed evidence has been collected regarding the types of resistance to be expected under the respective targeted drug. The most common acquired alteration to first- and second-generation EGFR TKI is the EGFR T790M, which occurs in approximately 50% of cases [17]. Osimertinib is a third-generation TKI that covalently binds to the C797X residue in the ATP binding site of EGFR. Consequently, osimertinib is still effective in T790M-mutated tumors, and T790M does not occur in response to it [9]. The opposite has been observed quite frequently in tumors treated with osimertinib in second-line. A disappearance of the T790M, also referred to as “loss of T790M” occurs in about 50% of cases, including our patient [10,18,19]. The term “loss of T790M”, may be misleading since it suggests reconstitution of the T790M variant back to wildtype. However, the loss probably occurs through suppression of the tumor clone carrying the T790M mutation. Some evidence suggests that subclonal T790M, that is, the presence of T790M only in a fraction of tumor cells, might negatively influence the response to osimertinib [20,21]. Another cause for T790M “loss” may lie in the often subclonal nature of T790M combined with technical limitations, as the allele frequency of T790M may lie below the assays validated lower limit of detection (LOD) [21]. In our case, at both timepoints, T790M was not detectable, not even as isolated alleles below the LOD. Still, we cannot exclude a subclonal distribution of T790M responsible for reducing T790M relative to L858R. Nevertheless, twice during the course of the disease, firstly after quadruple carboplatin/paclitaxel/atezolizumab/bevacizumab and later after pemetrexed/cisplatin chemotherapy, the T790M was undetectable, providing a rationale for TKI re-induction. During the course of the disease, two new TP53 mutations were detectable in the liquid biopsy. Data, however, indicate that TP53, similar to driver mutations EGFR, MET, and BRAF, are usually clonal events and therefore should be expected to be present at primary diagnosis [22]. Therefore, in our case we cannot rule out the possibility of tumor heterogeneity, subclonal manifestation of *TP53* mutations, or even a second primary tumor as a source of *TP53*-mutated ctDNA.

Liquid biopsy constitutes a minimally invasive alternative to tissue-based testing. Due to the inherently reduced risk of complications, ctDNA testing offers the potential to monitor the disease at multiple timepoints during the course of the disease without the necessity of receiving tissue biopsies. At all timepoints, liquid biopsy revealed the original driver mutation *EGFR* L858R. On one occasion, the cobas^®^ test was performed instead of the HC NGS due to the limited DNA concentration. This PCR-based assay does not indicate the allele frequency of the found variants, and it lacks other genes like *TP53* that might be helpful in understanding tumor dynamics and complexity. Especially in a late-stage disease with elevated tumor burden and difficult-to-access metastases in bone or brain, liquid biopsy can be helpful in obtaining adequate amounts and quality of analyte. CtDNA analysis, however, is not trivial and should be performed by experienced laboratories. Limitations should be clearly stated in the molecular pathological report, including LOD, AF, type of assay, and coverage of the genetic area. An all-wildtype report should discuss the possibility of a false negative result. A liquid biopsy assay should ideally provide a LOD below 1%. This has been shown to enable the detection of somatic alterations in more than 70% of cases resulting from liquid biopsy and even higher rates (>80%) for select genes like *EGFR* [23,24].

The appearance of C797S in our case opposes further targeted therapy approaches. In the AURA trial, NGS analysis of ctDNA from 73 patients that progressed after second-line treatment with osimertinib revealed 14% of C797S, compared to 7% in FLAURA (first-line osimertinib) [7,8]. The medical need in these patients is determined by the reduced response to immuno-oncology of *EGFR*-positive tumors and by the insensitivity of C797S to practically all third-generation *EGFR* TKIs, including rociletinib, olmutinib, lazertinib, and abivertinib [11]. In our patient, the allelic context of resistance mutations T790M and C797S is cis-structured, indicating location on the same allele. Although cis configuration reflects the more common configuration type (85%) compared to trans, it is not responding to a combination of first- and third-generation TKI [25,26]. For “in cis” mutated tumors, a combination of brigatinib (dual *EGFR* and *ALK* inhibitor) plus anti-*EGFR* antibody (i.e., cetuximab, panitumumab) has shown in vitro activity against triple mutant cells with T790M/C797S and has led to a phase I/II trial [27,28]. A potentially more targeted approach is prospectively evaluated in the SYMPHONY phase I/II trial, investigating the fourth-generation *EGFR* TKI BLU-945 as monotherapy or in combination with osimertinib in C797S mutated NSCLC [29]. Other next-generation small molecules (i.e., HS-10375) are also subject to clinical trials [30]. These options are currently being evaluated and should add to the rationale for molecular testing at the time of progression to *EGFR* TKI in the future. Outside of clinical trials and in the absence of druggable driver mutations, further line treatment after progression to osimertinib should include chemotherapy. While the addition of the PD-1 antibody pembrolizumab to platinum-based chemotherapy was not superior to chemotherapy alone (KEYNOTE-789 trial), the addition of both bevacizumab, an anti-VEGF antibody and the PD-L1-based immune checkpoint inhibitor atezolizumab showed promising outcome results with a median overall survival of 29.4 months in the ImPOWER150 trial [31,32]. The patient in our case report was also successfully treated by this four-drug combination for many months. All lines of therapy were tolerated well, and side effects did not exceed common toxicity criteria grade 2. There was no delay in therapy in any line due to side effects or poor tolerance.

In summary, we could show that plasma-based ctDNA testing is feasible in clinical practice and can be useful in tracking primary and acquired molecular alterations to adjust treatment strategies. A loss of the T790M-carrying clone was observed after osimertinib failure at two timepoints during the course of therapy and allowed for re-induction with erlotinib and osimertinib, respectively, resulting at least in a transient response. At the time of preparing this manuscript, the patient had achieved seven-year therapeutic success by alternating IO/chemo and targeted therapy based on longitudinal ctDNA analysis. Sequential liquid biopsy testing by NGS is usually not covered by health care providers.

## Data Availability

Data sharing is not applicable to this article.
